# A Systematic Review of Medical Treatments for Benign Prostatic Hyperplasia in Dogs: Evaluating Strategies for Reproductive Function Preservation

**DOI:** 10.3390/vetsci12010070

**Published:** 2025-01-19

**Authors:** Florin Petrișor Posastiuc, Nicolae Tiberiu Constantin, Guillaume Domain, Ann Van Soom, Alexandru Ilie Diaconescu, Mario Darius Codreanu

**Affiliations:** 1 Department of Internal Medicine, Reproduction and Population Medicine, Faculty of Veterinary Medicine, Ghent University, 9820 Merelbeke, Belgium; florin.posastiuc@ugent.be (F.P.P.); guillaume.domain@ugent.be (G.D.); ann.vansoom@ugent.be (A.V.S.); 2 Department of Clinical Sciences II, Faculty of Veterinary Medicine, University of Agronomic Sciences and Veterinary Medicine of Bucharest, 050097 Bucharest, Romania; alexandru.diaconescu@fmvb.usamv.ro (A.I.D.); mario.codreanu@fmvb.usamv.ro (M.D.C.)

**Keywords:** benign prostatic hyperplasia, breeding dogs, fertility preservation, sperm quality, medical therapy

## Abstract

Benign prostatic hyperplasia (BPH) is a common condition in intact male dogs and a leading cause of subfertility. While surgical and chemical castration are effective treatments for BPH, managing this condition in breeding dogs is challenging due to the desire to preserve reproductive function. This study systematically reviews medical treatments for BPH that maintain breeding potential. Thus, a comprehensive literature search was conducted across four databases (CAB Direct, Scopus, PubMed, and Web of Science) to identify peer-reviewed studies published between 2000 and 2024, following PRISMA guidelines. Specific inclusion and exclusion criteria were applied, and a risk of bias assessment was performed using a modified Cochrane tool. A total of 35 studies were included, evaluating therapies such as finasteride, osaterone acetate, tamoxifen, and others, with outcomes including prostate size, sperm quality, hormonal levels, and tissue morphology. Evidence suggests that osaterone acetate and finasteride are acceptable treatments for BPH in breeding dogs, in spite of a still debatable effect on some sperm characteristics. Therapies like tamoxifen and acyline should be excluded due to their detrimental effects on reproductive function. Further research is required for apparently promising approaches concerning tadalafil, anastrazole, mepartricin, and *U. fissa*.

## Simple Summary

Benign prostatic hyperplasia (BPH) is a condition where the prostate gland enlarges in intact male dogs, which can lead to fertility issues. While surgical or chemical castration are effective ways to treat BPH, they are often unsuitable for breeding dogs because they can permanently or temporarily cease reproductive capabilities. This study reviews medical treatments for BPH that aim to maintain the dog’s ability to breed. To achieve this, research from the last 24 years was evaluated for both the quality and relevance of the delivered information. Results indicate that finasteride and osaterone acetate are the most studied molecules and suitable for treating BPH in breeding dogs, though their effect on sperm quality is still debatable. Other treatments, such as tamoxifen or acyline were shown to negatively impact reproductive function or sperm quality and are not recommended for breeding dogs. Additional options, such as tadalafil, anastrazole, mepartricin, and plant-based treatments like *Urtica fissa* extracts, show promising results but need further research to confirm their effectiveness. This study provides veterinarians with essential information for treating BPH in breeding dogs while aiming to preserve fertility.

## 1. Introduction

Benign prostatic hyperplasia (BPH) is the most common prostatic disease in dogs [1,2,3], with approximately 50% of intact males showing histological signs of BPH by 4 years of age and over 90% becoming affected by eight years of age [4]. The most prominent feature of BPH is prostate enlargement, resulting from a combination of hypertrophic and hyperplastic mechanisms [5]. Hypertrophy, characterized by an increase in cell size, is primarily estrogen-induced [6,7]. In contrast, hyperplasia involves an increase in cell numbers [6]. This mechanism plays a more significant role, as increased cellular proliferation is fundamental to the prostate size enlargement observed in BPH [8]. The etiopathogenesis underlying the hyperplastic events is attributed to a metabolic shift involving testosterone [9]. Specifically, this shift is driven by the marked conversion of testosterone to dihydrotestosterone (DHT) through the enzymatic action of 5 α-reductase [1,10]. However, estrogen and testosterone work in concert to influence overall prostate growth [7]. Estrogens can contribute to an increase in the number of androgen receptors [4,11] and potentially stimulate prostate growth by promoting stromal cell proliferation [12].

Although often asymptomatic, BPH can cause moderate to severe clinical issues [3]. These include hematospermia, hematuria, dysuria, constipation, and tenesmus [11,13]. In more advanced cases, prostate enlargement can lead to complications such as prostatitis, cysts, and perineal hernias [3]. In dogs used for reproduction, commonly referred to as breeding dogs, BPH is particularly concerning due to its association with subfertility or even infertility, with recent literature emphasizing the role of acquired factors in fertility drops in dogs [4,14,15]. One study reported that 32.8% of infertile dogs were diagnosed with BPH [14]. The direct effect of BPH on semen quality often translates into increased DNA fragmentation and a higher incidence of morphological defects [4]. These issues are linked to the characteristic oxidative status of the hyperplastic prostate [16,17,18]. In BPH, reactive oxygen species accumulate, contributing to oxidative stress and decreasing local antioxidant defenses [19]. This imbalance extends to the prostatic fluid, which constitutes a significant portion of the seminal plasma and is in direct contact with sperm cells [20]. Thus, the altered biochemical environment of the prostatic fluid exacerbates oxidative damage, with sperm cells being particularly susceptible to oxidative stress, a factor that further contributes to the observed decline in semen quality [18,20,21].

While surgical and chemical castration are commonly preferred options for managing BPH in non-breeding dogs [22,23], these approaches are often considered unsuitable for this patient category, where preserving fertility is a priority. A diverse array of medical therapies has been used to alleviate symptoms and reduce prostatic volume; however, their effects on reproductive function may vary depending on the used therapeutic agent. This systematic review aims to rigorously assess the suitability of medical treatments for BPH in breeding dogs, with particular emphasis on evaluating secondary effects and identifying strategies that prioritize the preservation of sperm quality.

## 2. Materials and Methods

A comprehensive literature search was conducted across four major databases: CAB Direct, Scopus, PubMed, and Web of Science, following the Preferred Reporting Items for Systematic Reviews and Meta-Analyses (PRISMA) guidelines [24]. The search strategy was designed to identify relevant studies on BPH in dogs investigating various treatments that do not cease reproductive activity. Therefore, papers addressing surgical or chemical castration were excluded. Search queries were conducted using a combination of terms relevant to BPH and its treatment strategies as defined in the Supplementary Materials (Table S1). Moreover, the same search query was tailored to each database by incorporating Boolean operators (AND, OR, NOT), MeSH terms for PubMed, and specific filters, indexing terms, or citation indexes for the other three resources. The preliminary queries were limited to titles, abstracts, and keywords and included only peer-reviewed studies published in English. A timeframe restriction from 2000 to 2024 was applied. Further inclusion criteria were defined in Table 1. The search on all databases was carried out on 25 September 2024.

A secondary screening was conducted on the full-text versions of the selected papers. To minimize bias, a second reviewer independently screened the studies. Any disagreements were resolved through discussion, or by consulting a third reviewer if necessary. Following this process, some manuscripts were excluded (*n* = 3), with the reasons for exclusion detailed in Table S2. Papers that proceeded to the full data extraction stage (*n* = 35) are summarized in Table S3, which provides an overview of the therapies, measured outcomes, methods employed, and participant numbers for each study.

In addition, the methodology included a risk of bias assessment using a modified version of the Cochrane Risk of Bias tool, following the approach of Charalambous et al. 2014 [25]. Each study was evaluated across six domains: random sequence generation, allocation concealment, blinding of participants, personnel and outcome assessors, completeness of outcome data, and selective reporting of outcomes. Each domain was assigned a numerical score: high risk of bias = 3, moderate or unclear risk of bias = 2, and low risk of bias = 1. The total score for each study was calculated by summing the scores across all domains, which classified the overall risk of bias as follows: Score 19–21 = high risk; 16–18 = moderate/high risk; 13–15 = moderate risk; 10–12 = low/moderate risk; 7–9 = low risk (Table S4).

The authors adhered to the PRISMA 2020 checklist in writing this paper.

## 3. Results

**Figure 1:**
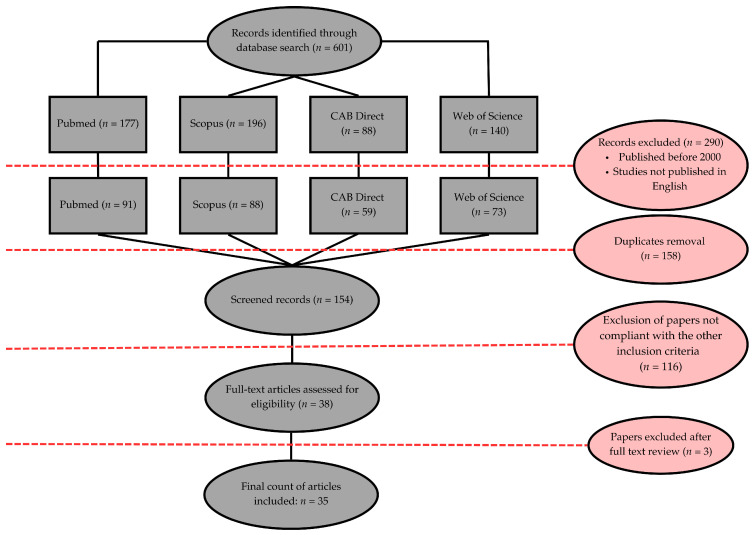
Preferred Reporting Items for Systematic Reviews and Meta-Analyses (PRISMA) flow diagram of study selection process.

A total of 601 records were initially identified through database searches: PubMed (177 records), Scopus (196 records), CAB Direct (88 records), and Web of Science (140 records). After excluding 290 records published before 2000 or not in English, 311 records remained. Duplicates were removed, leaving 154 unique records for screening. From these, 116 records were excluded based on non-compliance with inclusion criteria (e.g., review articles, case reports, studies not involving dogs, studies lacking a focus on medical treatments, or unavailable full texts). This process resulted in 38 articles being assessed for full-text eligibility. After further review, three additional papers were excluded, leading to a final inclusion of 35 studies in the analysis. The literature selection process is resumed in Figure 1.

### 3.1. Evaluated Therapies

**Figure 2:**
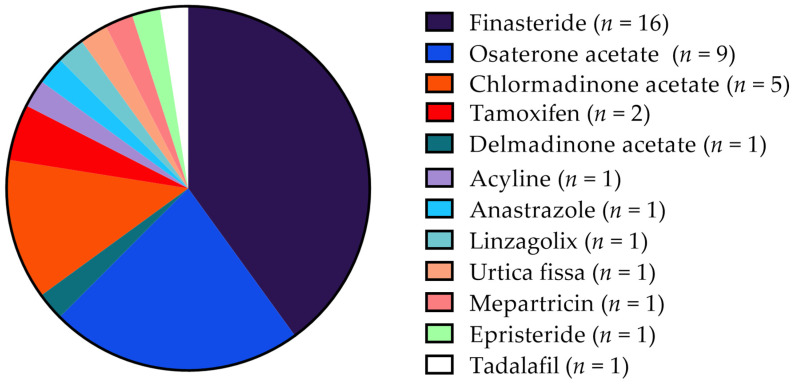
Visual overview of therapies evaluated in the reviewed studies.

The therapies evaluated in the selected study include finasteride [9,26,27,28,29,30,31,32,33,34,35,36,37,38,39,40], epristeride [40], osaterone acetate [26,41,42,43,44,45,46,47,48], delmadinone acetate [26], chlormadinone acetate [36,49,50,51,52], tamoxifen [53,54], acyline [55], anastrazole [54], linzagolix [56], *U. fissa* [39], tadalafil [57] and mepartricin [58] (Figure 2). Sixteen studies assessed the effect of finasteride, comparing results with control groups and other treatments assessed either within the same studies or cited in the literature. Osaterone acetate was included in nine trials, whereas other treatments were evaluated in fewer instances: chlormadinone acetate (*n* = 5), tamoxifen (*n* = 2), delmadinone acetate (*n* = 1), acyline (*n* = 1), linzagolix (*n* = 1), mepartricin (*n* = 1), tadalafil (*n* = 1), anastrazole (*n* = 1), epristeride (*n* = 1), and *U. fissa* (*n* = 1).

### 3.2. Measured Outcomes

**Figure 3:**
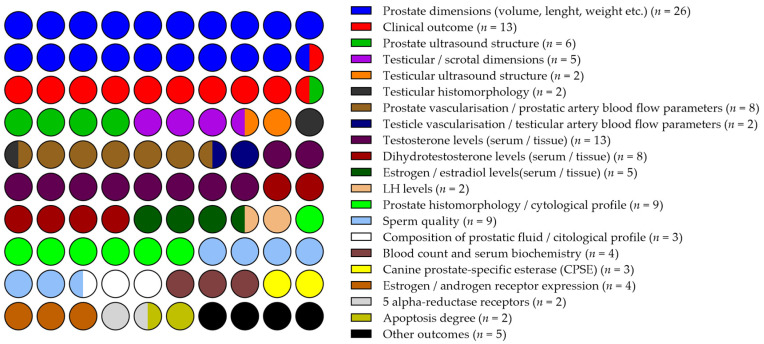
Overview of the outcomes assessed in the reviewed studies.

The studies included in this review assessed a diverse range of outcomes related to the effects of BPH pharmacotherapies (Figure 3). Most investigations evaluated changes in prostate dimensions [26,27,28,30,31,32,33,34,35,36,38,39,41,44,45,46,47,48,49,51,52,53,54,55,56,59] and clinical outcomes [9,26,28,35,38,44,45,46,48,53,54,55,59]. Prostatic and/or testicular ultrasonographical appearance was assessed in six papers [41,43,45,53,54,55,59], while prostate vascularization, measured through prostatic artery blood flow or tissue perfusion metrics, was examined in eight studies [27,31,32,33,43,45,55,59]. Testicular or scrotal size [9,43,49,53,54] and testicular histomorphology [9,49] were assessed in several studies. In addition, testicular vascularization and tributary artery blood flow parameters were included in two studies [9,43]. Hormonal changes were analyzed in fourteen setups, focusing on parameters such as testosterone [9,28,30,36,38,40,44,47,48,53,56,57,59], DHT [9,28,30,36,38,40,57,59], estrogen/estradiol [9,28,36,44,58] and LH levels [47,48].

Histomorphological or cytological profiles of the prostate were included as follow-up outcomes in nine studies [28,34,36,39,50,51,52,58,59], while the composition or cytological features of prostatic fluid were described before and after treatment in only three studies [37,41,42].

Several studies [9,29,35,38,41,42,47,53,54] reported the effects of treatment on sperm quality, mentioning standard features such as fractional volumes, concentration, total sperm count, motility, viability, and sperm morphology. Kinematic parameters, plasma membrane and acrosome integrity, mitochondrial activity, DNA integrity, sperm binding capability, and susceptibility to oxidative stress were included in only three experimental designs [9,29,42].

Blood counts and serum biochemistry parameters were evaluated in three studies [30,44,54,57]. Additionally, some publications [28,30,36,45,49,50,51,52,58,59] included other potential markers such as the canine prostate-specific esterase (CPSE) [30,45,57], prostate-specific antigen (PSA) [30,57], acid phosphatase [30,57] along with the expression of 5 α -reductase type I and type II receptors [50,52], estrogen and androgen receptor expression [49,50,52,58], apoptotic index/degree of apoptosis [36,51], vascular endothelial growth factor (VEGF) [28] and caspase-3 [59].

### 3.3. Methods Applied for Outcome Measurement

**Figure 4:**
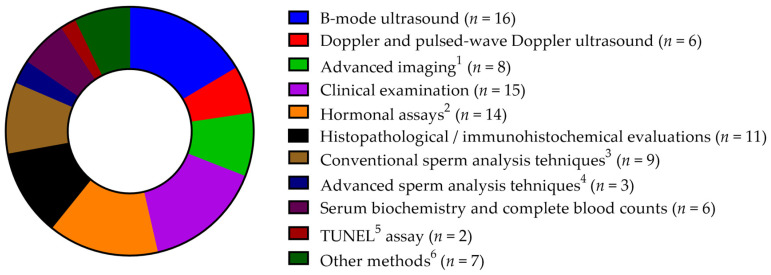
Overview of the methods used in the reviewed studies.

A diverse range of methodologies was employed across the reviewed studies to evaluate outcomes of interest (Figure 4).

Footnotes:
^1^ contrast-enhanced ultrasound, radiographical examination following retrograde urethrocystography, magnetic resonance imaging (MRI) and computed tomography (CT)
^2^ radioimmunoassays (RIA) and enzyme-linked immunosorbent assays (ELISA)
^3^ phase contrast microscopy for subjective motility evaluations, manual sperm counting, direct morphology and viability assessments (using Diff-Quick, Rose-Bengal, Eosin-Nigrosin or Bydgoska stainings)
^4^ computer assisted sperm analysis (CASA), thiobarbituric acid reactive substances (TBARS), oxidation of 3′3 diaminobenzidine by cytochrome-C (DAB), modified blue toluidine staining, flow cytometry, sperm binding test in the perivitelline membrane
^5^ terminal deoxynucleotidyl transferase dUTP nick end labeling
^6^ gas chromatography-mass spectrometry (GS-MS), bacterial cultures, quantitative polymerase chain reaction (PCR), flame photometry and colorimetric assays

Imaging techniques were prominently used, with B-mode ultrasound frequently assessing organ size and morphology [9,26,27,28,30,35,36,38,41,43,45,46,53,54,55,59]. Doppler and pulsed-wave Doppler ultrasound were applied in several studies to measure blood flow and vascularization in the prostate, testicles, and associated arteries [9,27,28,43,55,59]. Advanced imaging methods, such as contrast-enhanced ultrasound, radiographical examination following retrograde urethrocystography, magnetic resonance imaging (MRI), and computed tomography (CT) scans, were also reported [31,32,33,34,38,39,45,47].

Clinical evaluations were widely used, with many studies incorporating physical examinations to assess treatment efficacy [9,26,28,35,38,39,41,44,45,46,48,53,54,55,59].

Hormonal analyses employed radioimmunoassays (RIA) and enzyme-linked immunosorbent assays (ELISA) to quantify testosterone, DHT, and other hormones relevant to BPH [9,28,30,36,38,40,44,46,47,48,53,57,58,59].

Histological assessments, including immunohistochemistry and various staining techniques, provided valuable insights into treatment-induced changes in tissue morphology and cellular characteristics [9,28,34,36,39,49,50,51,52,58,59].

Sperm quality assessments were common, using both conventional and advanced methodologies. Conventional techniques included phase contrast microscopy for subjective motility evaluation, manual sperm counting, morphology, and viability assessments using stains such as Diff-Quick, Rose-Bengal, Eosin-Nigrosin, and the Bydgoska method [9,29,35,38,41,42,47,53,54]. Advanced methods employed computer-assisted sperm analysis (CASA), thiobarbituric acid reactive substances (TBARS), cytochrome-c oxidation of 3′3-diaminobenzidine (DAB), modified toluidine blue staining, flow cytometry, and sperm binding tests [9,29,42].

Serum biochemistry and complete blood count analyses were applied in six studies to assess systemic health impacts [30,38,44,53,54,57]. Apoptosis detection and quantification were conducted using the terminal deoxynucleotidyl transferase dUTP nick end labeling (TUNEL) assay [36,37]. Additional methods reported less frequently included gas chromatography-mass spectrometry (GS-MS) [36,48], bacterial cultures [35], quantitative polymerase chain reaction (PCR) [28], flame photometry [30] and colorimetric assays [30,41,57].

### 3.4. Quality of Studies

**Figure 5:**
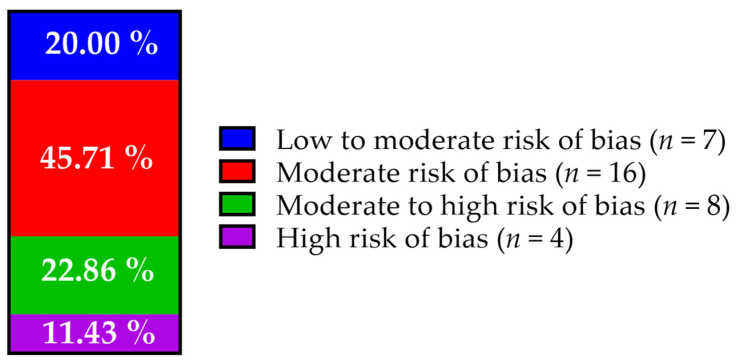
Distribution of overall risk of bias across included studies.

The systematic bias assessment revealed concerns regarding the methodological rigor of several studies, particularly due to insufficient reporting of randomization sequence generation and allocation concealment. These elements are crucial for minimizing bias in group assignments, and their inadequate description can compromise the internal validity of the studies and the reliability of conclusions about treatment effectiveness. Nonetheless, the majority of included studies (*n* = 23) demonstrated a low to moderate or moderate risk of bias. In contrast, eight studies were classified as having a moderate to high risk, and four were identified as having an overall high risk of bias (Figure 5). A detailed analysis of the bias assessments, along with the rationale for point allocation across each criterion, is provided in Table S4.

## 4. Discussion

This systematic review evaluated the efficacy of available pharmacotherapies for BPH in breeding dogs, with a specific focus on treatments that preserve reproductive functionality. The findings indicate that a range of options is available, but their effectiveness and potential side effects, including their impact on fertility, vary.

### 4.1. Finasteride

Finasteride is a competitive 5 α-reductase inhibitor that does not bind to the androgen receptor [60,61]. This characteristic may account for its relatively lesser impact on reproductive function and sperm quality, as previously noted [38]. Within the revised literature finasteride was used in spontaneous BPH dogs or canine BPH models with protocols varying between 0.1 mg/kg to 1 mg/kg SID [9,26,27,28,29,30,31,32,33,34,35,36,37,38,39,40]. Dose regimens ranging from 0.1 to 0.5 mg/kg SID caused no life-threatening adverse reactions, and no significant differences in blood counts or serum biochemistry after 14 or 28 days of treatment were observed [30], nor after 16 weeks of treatment [38].

Clinically, most studies reported resolution of BPH symptoms within 30 days of treatment with finasteride, using 0.1 to 0.5 mg/kg SID [35,59], or a standard fixed dose of 5 mg per dog SID [9,28]. However, blood may continue to be present in the ejaculate, as it was observed after both 60 [59] and 112 days of treatment [35,38].

Finasteride appeared to effectively reduce prostate size, though the degree and rate of reduction vary with dosage and duration of treatment [27,28,31,33,35,36,38,39,59]. The reduction in prostate volume was typically not immediate, highlighting the slow-acting nature of finasteride. Namely, significant volume changes between treated and untreated BPH cases only became apparent after 60 days of treatment when a standard dose of 5 mg/animal SID was used [27]. This delayed effect aligns with findings from another study, where a significant reduction in prostate volume was only evident at the 60-day mark, with the prostate reaching the expected size in relation to the dog’s body weight [28]. In a study utilizing a higher and weight-adjusted dosage of 1 mg/kg SID, a more rapid therapeutic effect was observed, resulting in a 35% reduction in prostate volume after a four-week treatment period [33]. Furthermore, the same dosage resulted in an even more substantial reduction in prostate size, achieving a 47–54% decrease after 12 weeks of treatment [31,33]. In contrast, lower dosing regimens (0.1–0.5 mg/kg SID) produced more variable outcomes. For instance, one study reported a 34.02% reduction in prostate volume after four weeks of treatment [35], while another study indicated only a 17.1% reduction with a 0.5 mg/kg dose and a 13.6% reduction with the lower 0.1 mg/kg dose over the same four-week period [59]. Greater discrepancies in outcomes were observed while reporting on eight-week treatment durations with the 0.1–0.5 mg/kg SID regimen. One study documented a 55.88% reduction in prostate size [35], whereas another reported volume reductions ranging from only 18.2% to 20.1% [59].

The effects of finasteride on prostate structure have been well-documented through both imaging [31,32,33,59] and histopathological studies [28,39,59]. Finasteride treatment generally leads to observable improvements in prostate imaging [59], although the extent of these improvements often depends on the baseline severity of abnormalities detected via ultrasound. One study has shown that cysts smaller than 0.5 cm in diameter tend to resolve after 60 days of finasteride treatment [59]. Despite these imaging-based improvements, the histological structure of the prostate appears minimally affected by finasteride, as treated and untreated BPH dogs exhibit comparable prostatic histological features [28,59]. Only one paper stated that finasteride was able to induce the inhibition of glandular epithelium proliferation [39]. More consistent changes were reported in prostatic vascularization [27,31,32]. Using dynamic contrast-enhanced MRI, finasteride was shown to affect perfusion dynamics in the prostates of dogs with BPH [31,32], slowing tissue perfusion, likely due to alterations in permeability rates [31]. During the clinical progression of BPH, the peak systolic to diastolic velocity (S/D) typically decreases [62]. Interestingly, finasteride treatment can increase the S/D in dogs affected by BPH, as demonstrated in a study where treated dogs had a higher S/D than untreated BPH dogs [27,59].

Finasteride treatment significantly reduced DHT levels in both blood serum [28,30,38,40] and prostatic tissue [36,40], with minimal impact on serum testosterone levels [28,30,38], except for one study reporting an increase [40]. However, results regarding testosterone variation should be interpreted cautiously, as none of the cited studies included stimulation with GnRH or hCG in their experimental designs.

Considerably high values of serum CPSE are associated with BPH [63]. The impact of finasteride treatment on this marker is notable, as it induces a significant reduction in CPSE serum levels, similar to its effect on PSA [30].

In terms of fresh sperm quality, finasteride treatment did not cause significant detrimental effects, but it also did not result in substantial improvements. Thus, the percentage of progressively motile sperm and morphologically normal sperm [35,38] or living spermatozoa [35] did not significantly change after treatment. Semen volume was the only negatively impacted parameter [35,38], a finding of limited practical importance, as total sperm counts remained unchanged [35]. Additionally, the reduction in semen volume was not sustained beyond 16 weeks of treatment [38]. The effects of finasteride on sperm kinematic parameters are diverse, with one study indicating no impact [9], while another suggested that finasteride may lower average sperm velocities [29]. Moreover, frozen-thawed semen from dogs with BPH receiving finasteride showed lower sperm velocity, curvilinear velocity, and amplitude of lateral head displacement compared to cryopreserved sperm from untreated BPH dogs [29]. This could be related to the higher number of apoptotic cells ejaculated by dogs receiving finasteride [37]. While this observation may reflect a healthy rate of cell turnover within the prostate in response to treatment, it also suggests that a more stringent separation of sperm fractions is necessary when attempting cryopreservation of sperm from treated dogs. On the other hand, there is a positive effect of the treatment on semen freezeability, with finasteride causing superior DNA integrity and sperm binding capacity [29].

One big inconvenience of the use of finasteride is that the owner needs to comply with daily treatment. In one study [34] crosslinked polyvinyl alcohol nanofibrous particles were used as drug delivery systems for finasteride through a minimally invasive technique used in the treatment of BPH in men called prostatic artery embolization [64]. A high efficiency in reducing prostate volume was demonstrated, with no embolization observed in the periprostatic tissues of dogs suffering from BPH [34]. This showed a consistent reduction in prostatic volume even 6 months after the procedure [34]. However, there is no data on the possible impact that this method may have on sperm quality or fertility in dogs.

The use of finasteride in BPH-affected dogs has not been associated with severe side effects or significant negative impacts on general health. Two separate studies monitored blood serum biochemical parameters and complete blood counts for potential alterations caused by the treatment [30,38]. One study, after 16 weeks of finasteride use, found no remarkable differences [38], while another, after one month, observed negligible variations in most hematological or biochemical factors [30]. When significant changes were detected, they remained within physiological reference ranges [30]. However, since these assessments focused on relatively short-term finasteride use, caution is still advised for long-term treatment, as potential adverse effects cannot be entirely excluded.

### 4.2. Epristeride

Epristeride is a noncompetitive 5α-reductase inhibitor shown to reduce prostate size and relieve symptoms in men suffering from BPH [61]. Only one study has evaluated the effects of epristeride in dogs with BPH, comparing its impact on testosterone and DHT levels with that of finasteride [40]. While epristeride demonstrated similar effectiveness to finasteride in lowering both serum and prostatic DHT levels, its distinct receptor affinity results in a comparatively smaller impact on testosterone levels [40]. This difference suggests that epristeride may have a reduced influence on spermatogenic processes. However, the effects of epristeride on other outcomes in BPH dogs remain unknown.

### 4.3. Osaterone Acetate

Osaterone acetate is a steroidal antiandrogen [65], commonly used in dogs for BPH treatment at a dosage ranging from 0.25 to 0.5 mg/kg SID, for consecutive days [26,41,42,43,44,45,46]. Alternative dosing regimens, including 0.1, 0.2, and 1 mg/kg SID, have also been investigated [47,48]; however, these dosages did not become established as standard protocols.

The therapeutic effects of osaterone acetate are noted rapidly, with substantial improvements in clinical symptoms observed shortly after the seven-day treatment ends [46]. In one study [26], half of the dogs treated with 0.25 mg/kg SID experienced complete clinical recovery within two weeks. Interestingly, another study reported an even faster effect, with 80% of subjects reaching full remission by the end of the seven days of treatment [44]. In fact, overall remission rates are notably high, ranging from 82.6% to 100% among dogs following the standard seven-day protocol (0.25–0.5 mg/kg SID) [26,44,46]. Generally, the typical timeframe for complete remission extends from 14 to 28 days after treatment initiation [44,45,46]. However, after administering 0.25 mg/kg SID, one study found that 84% of BPH-affected dogs became symptom-free only 180 days after the initial drug administration [26]. In addition, other reports indicate that if a lower dose is desired (0.1–0.2 mg/kg), the overall effect of the therapy may be delayed [47,48].

Prostatic volume reduction has been demonstrated in several studies [41,43,45,47]. A significant decrease may be observed as early as 14 days after the initiation of treatment [43], with a 57–64.3% decrease at 60 days, as assessed by B-mode ultrasound [41,46]. Even if a complete recovery of pretreatment prostatic size may be observed only after six months [47], retreatment is likely to be required earlier to prevent the recurrence of severe clinical symptoms. Most optimistic reports state that more than 80% of subjects receiving osaterone acetate remained in remission over a six-month period [26]. Overall, the suggested relapse rate varies between 16.1–40% at 168–169 days post-treatment [26,42]. Additionally, other findings indicate that the reduction in prostate volume may last only about 20 weeks [43], with evidence of regrowth appearing earlier; for instance, prostates were 12% larger at 180 days post-treatment initiation compared to measurements taken at 120 days [41]. Thus, detecting prostate regrowth via ultrasound could facilitate early intervention to prevent the recurrence of clinical symptoms and may indicate the necessity for retreatment.

The induced volumetric reduction is accompanied by vascular changes [43,45]. Specifically, reduced tissue perfusion was observed 21 days after treatment initiation [45]. Moreover, certain blood flow parameters, such as peak systolic velocity and mean velocity measured in the prostatic artery, decreased under the effect of standard therapeutic dosages [43].

Osaterone acetate has the capacity to produce further improvements in ultrasonographic structure [43,45]. Approximately 60% of dogs showed a more homogeneous prostate, with decreased echogenicity noted in 40% of the treated dogs [43]. Additionally, if prostatic cysts or other focal lesions were identified before treatment, they either decreased in volume [45] or became undetectable in 60% of cases by eight weeks post-treatment [43]. Cyst resolution was observed, particularly when the structures did not exceed 10 mm in diameter [43].

The impact of the treatment on serum testosterone levels appears to be temporary. Studies that measured this parameter consistently reported an initial decrease in testosterone levels during therapy, followed by a return to baseline values within 2 to 3.7 months post-treatment [44,47,48]. Furthermore, a significant decrease in CPSE serum levels was registered 21 days after the start of the treatment [45].

In terms of sperm quality, several transient changes have been reported, though the overall impact of osaterone acetate remains debatable. The primary findings indicate that osaterone acetate may affect sperm morphology, notably increasing tail defects [41]. One study [41] reported a 20% reduction in morphologically normal sperm 60 days after the start of the treatment, with morphology returning to baseline within 45 to 180 days [41,47]. In contrast, another study found no significant changes in sperm morphology but noted a temporary decrease in the percentages of motile and progressively motile sperm, significant only at 21 days and not persisting at 8 or 12 weeks post-treatment [42]. Moreover, a reduction in the third fraction volume was also observed, along with an increase in semen concentration eight weeks after therapy [42]. Interestingly, between 120 and 180 days post-treatment, a temporary decrease in Zn^2^⁺ levels in the prostatic fraction was highlighted, but spontaneously normalized by day 240 [41].

Several general health adverse reactions were reported with the use of osaterone acetate [26,44,46], despite no significant changes being observed in complete blood counts or serum biochemical analyses during treatment [44]. Increased appetite was noted in three studies [26,44,46], resulting in weight gain in 40% of the treated dogs [44]. Apathy was observed in 13% of dogs in one study [46]. Other side effects included transient polyuria/polydipsia [44], gastrointestinal symptoms [44] such as vomiting or diarrhea [26], transient behavioral changes [26], decreased activity levels [44], and dry or dull coat [44].

### 4.4. Chlormadinone Acetate and Delmadinone Acetate

Chlormadinone acetate and delmadinone acetate belong to the pharmacological class of progestogens, which includes other compounds such as megestrol acetate and medroxyprogesterone acetate. However, while megestrol acetate and medroxyprogesterone acetate were investigated for the treatment of BPH several decades ago [66,67], interest in their use for this purpose has not remained consistent. In contrast, reports on the use of chlormadinone and delmadinone acetate for managing BPH in dogs continued to be published after 2000 [26,36,49,50,51,52], reflecting sustained interest in these compounds for veterinary applications over the past two decades.

Both chlormadinone acetate and delmadinone acetate are synthetic steroidal antiandrogens [49,68] that have proven effective in treating dogs with BPH [26,36,49,50,51,52]. The actions of these molecules result in a reduced intake of testosterone in the prostate gland, explained by their selective interaction with the androgen receptor, manifested through competitive binding [69]. This mechanism was specifically investigated in BPH dogs receiving chlormadinone acetate at doses ranging from 0.03 to 0.3 mg/kg/day for 6 months [49,50,52].

The dose regimen appears to significantly affect the impact of chlormadinone acetate, leading to varying degrees of prostate atrophy correlated with the degree of interaction with the androgen receptor, as demonstrated by immunohistochemistry [49,50,52]. Despite this dose-dependent effect, chlormadinone acetate was able to produce an overall shrinkage in both prostatic compartments—glandular epithelium and stroma—regardless of the dose [52]. This effect led to a significant overall decrease in prostate size and weight [36,49,50,52]. The higher dose (0.3 mg/kg/day) resulted in a 30% reduction after only five weeks of treatment, similar to findings in BPH dogs receiving finasteride in one study [36].

By reducing the intracellular uptake of testosterone, chlormadinone targets prostatic tissue apoptosis [36]. The dose-dependent effect of chlormadinone acetate was further explained by the sequence of apoptotic processes that occur within the prostate during treatment. The apoptotic index was higher in low-dose treatments compared to higher-dose ones [51]. This finding was attributed to the prolonged duration of apoptosis in low-dose treatments, resulting in a slower regression in prostatic size [51].

Delmadinone acetate was administered to dogs via intramuscular or subcutaneous injection at a single dose of 3 mg/kg, resulting in a faster therapeutic effect than observed in studies using chlormadinone acetate [26]. Delmadinone acetate produced a 21% reduction in prostate volume within 14 days post-treatment, with volume reduction exceeding 35% by 60 days after administration [26]. Although its efficacy in prostate volume reduction at the 14-day mark was significantly lower compared to osaterone acetate, the clinical remission rate with delmadinone acetate was comparable to that achieved with osaterone [26]. Delmadinone acetate produced complete clinical recovery in 50% of treated dogs within two weeks, with recovery observed in 82.8% of cases by 180 days [26]. However, nearly one-fifth of the dogs experienced relapse after a mean period of 167 days, making retreatment necessary [26].

In terms of sperm production, histopathological examinations of tissue samples from dogs with BPH treated with chlormadinone acetate revealed no evidence of abnormal spermatogenesis or changes in Leydig cells [49]. Direct evidence of a temporary alteration of epididymal sperm maturation caused by the use of delmadinone acetate was stated in the abstract of one paper that could not be included in our analysis because the full-text version of the study was unavailable [70]. Overall, information regarding the impact of delmadinone acetate and chlormadinone acetate on sperm quality is limited. Therefore, their use in breeding dogs should be approached with caution.

Adverse reactions associated with the use of delmadinone acetate were reported in one study and were similar to those caused by osaterone acetate, occurring at comparable frequencies [26]. However, one severe side effect was noted as a dog developed irreversible hypoadrenocorticism [26].

### 4.5. Acyline and Linzagolix

Acyline and linzagolix are GnRH antagonists primarily used in human medicine [71,72,73], which have been tested in experimental studies on dogs with BPH [55,56]. In one study, a single administration of acyline at a dose of 330 μg/kg subcutaneously led to a mean reduction in prostate volume of 38.44%, with no adverse reactions reported throughout the trial [55]. Acyline effectively alleviated clinical symptoms, with BPH-related signs disappearing by day 15 post-treatment [55]. Ultrasound assessments showed significant improvements, including a reduction in prostatic echogenicity and the disappearance of cysts measuring 2–3 cm in diameter [55]. However, the therapeutic effects appeared to be relatively short-lived, lasting for less than 60 days, with peak efficacy observed around 30 days post-treatment [55]. By 60 days, all monitored outcomes including prostate volume, echostructure, and clinical signs returned to pre-treatment characteristics [55].

Linzagolix, an oral GnRH antagonist, was administered in one study at a dosage of 5 mg/kg SID [56]. Similar to acyline, linzagolix produced a 41% reduction in prostate weight after four weeks of treatment in BPH-affected dogs compared to controls [56]. In addition, serum testosterone levels consistently declined over the treatment period, reflecting linzagolix’s impact on androgen regulation [56].

Unfortunately, the direct effects of the two molecules on fertility and sperm quality have not been investigated in dogs affected by BPH. However, one study examining the effects of acyline in healthy, intact male dogs reported a drastic, though temporary, decline in semen quality, with two of the dogs in the study showing aspermia [74]. Reported effects on sperm parameters were decreased sperm concentration and motility, along with an increase in the percentage of morphologically abnormal sperm [74]. Conversely, another GnRH antagonist, antarelix, was found to significantly reduce total sperm output in stallions, although it did not affect sperm motility [75]. Therefore, it may be misleading to generalize the effects observed with one GnRH antagonist to another, as each may impact reproductive parameters differently. Additionally, potential species-specific effects should be considered. In light of all the discussed factors, it is advisable to avoid using a GnRH antagonist as a treatment for BPH in dogs intended for breeding during the treatment period.

### 4.6. Tamoxifen

Tamoxifen is a synthetic, nonsteroidal Type I antiestrogen compound that competitively blocks estrogen receptors and exhibits both antagonist and agonist effects [53]. The use of tamoxifen citrate as a treatment for BPH in dogs has been previously studied, with findings indicating that oral doses ranging from 0.16 to 0.42 mg/kg/day over 60 days [54] or 0.17 to 0.2 mg/kg/day over 28 days [53] can produce a relatively rapid reduction in prostate volume, noticeable already during the treatment period [53,54]. Prostate volume was observed to return to pretreatment values within five weeks after the completion of the regimen [53]. Moreover, tamoxifen was found to improve prostatic echogenicity and reduce the diameter of intraprostatic cysts [54], with no adverse effects reported in terms of clinical, hematological, or serum biochemistry findings [53,54].

In spite of these beneficial effects, tamoxifen should not be recommended for male dogs with BPH if breeding is intended. In two studies, tamoxifen led to a decrease in testicular width and consistency, along with a significant reduction in ejaculate volume, with some cases reporting aspermia throughout the treatment period [53,54]. Additionally, libido, sperm motility parameters, and total sperm counts were significantly reduced during treatment and up to two weeks afterward [53,54]. Semen morphology was also adversely affected [53].

### 4.7. Anastrazole

Anastrazole, a fourth-generation aromatase inhibitor primarily used in the treatment of infertility in men [76,77], has also been tested for the treatment of BPH in dogs [54]. Aromatase inhibitors reduce aromatase activity, thereby limiting the conversion of androgens into estrogens [78,79]. Therefore, the rationale for using such substances in the treatment of BPH lies in their ability to influence the estrogen-testosterone ratio. A 60-day regimen at oral doses ranging from 0.03 to 0.1 mg/kg/day resulted in a significant reduction in prostatic volume, with minimal volume achieved during the treatment period and sustained for one month post-treatment [54]. These results were achieved without affecting libido, testicular characteristics, or semen quality [54]. Additionally, no adverse clinical reactions were reported, and no significant changes were observed in hematological or serum biochemistry parameters [54]. Nevertheless, with only one relevant study available, choosing this molecule for treating BPH in breeding dogs should be approached cautiously.

### 4.8. Tadalafil

Phosphodiesterase-5 inhibitors have been investigated for their potential use in treating BPH in men [80]. Within this category, tadalafil showed promising results in a study focusing on dogs with induced BPH [57]. Similar to established BPH treatments like finasteride [30] and osaterone acetate [45], dogs with BPH that received oral tadalafil at 5 mg/day for 30 days showed a significant reduction in CPSE, PSA, and prostatic acid phosphatase serum levels [57]. Furthermore, the treatment did not cause any significant changes in blood count or serum biochemistry parameters in any of the treated dogs [57].

In men, daily administration of tadalafil may actually improve sperm quality [81]. However, little is known about its effects on sperm quality in dogs, and data on its direct clinical effects and potential for reducing prostate volume in dogs are lacking. Therefore, further investigation is necessary before considering tadalafil as a treatment option for canine BPH.

### 4.9. Mepartricin

Mepartricin is a semi-synthetic polyene macrolide complex produced by *Streptomyces aureofaciens* NRRL 3878 [82]. The pharmacotherapeutic approach for using mepartricin in treating human BPH is based on its capacity to lower circulating estrogen levels without affecting other hormones, such as androgens [83]. Despite androgen-dependent growth is central to the etiopathogenesis of BPH [4], the prostatic parenchyma is also responsive to estrogen levels [84]. In fact, estrogen may modulate the prostate’s sensitivity to androgens, likely by upregulating androgen receptor expression [4]. The beneficial effects of mepartricin in human BPH have been attributed to estrogen’s role in promoting the overgrowth of the fibromuscular component of the prostatic stroma [85,86].

In dogs with BPH, mepartricin showed similar effects [58]. Treated dogs exhibited reduced stromal proliferation scores, resulting in a significant decrease in prostate volume compared to controls [58]. The effects in dogs appear to be dose-dependent, with a dose of 20 mg/kg/day showing greater efficacy in reducing prostate size and significantly lowering both intraprostatic and serum estrogen levels [58]. Additionally, both estrogen and androgen receptor staining intensity were reduced with treatment, indicating clear effects on hormonal interactions [58]. However, there is insufficient information on the effect of mepartricin on sperm quality, libido, and fertility, as well as a lack of knowledge regarding its potential general health side effects, warranting further investigation before it can be considered an established treatment alternative for BPH in dogs.

### 4.10. U. Fissa

Species from the *Urtica* genus have been described as potential treatments for various conditions due to their numerous phytoconstituents, which may impact infectious processes or metabolic pathways [87]. In line with this, several herbal preparations from *Urtica dioica* extracts have shown 5-α-reductase inhibition, suggesting potential for treating BPH [88]. In dogs, a *U. fissa* polysaccharide fraction administered at 120 mg/kg/day for three months resulted in a 21% reduction in prostate volume [39]. Additionally, this regimen effectively inhibited glandular epithelium proliferation [39]. These findings suggest the potential for *Urtica* extracts in treating BPH, but further research is needed to assess their impact on sperm quality and clinical outcomes.

## 5. Conclusions

The medical management of BPH in breeding dogs continues to be a challenge, with osaterone acetate and finasteride being the most widely discussed treatments in the available literature. However, some aspects of these treatments, particularly regarding their effects on sperm quality, remain debatable after a thorough data review. Other therapeutic options, such as tamoxifen and acyline, are considered unsuitable for treating breeding dogs due to their detrimental impact on sperm quality. Caution is also warranted when considering delmadinone, chlormadinone acetate, or lizagolix, as their safety, efficacy, and direct effects on sperm characteristics in dogs have not been fully established. Additionally, treatments like tadalafil, anastrozole, mepartricin, and *U. fissa* extracts require further research to clarify their potential benefits and drawbacks. Overall, there is a critical need for more objective, well-designed studies on pharmaceutical treatments for BPH in breeding dogs.

## Figures and Tables

**Table 1:** Inclusion and exclusion criteria for studies on medical treatments that preserve reproductive function in male dogs with benign prostatic hyperplasia.

PICO ^1^ Framework	Inclusion Criteria	Exclusion Criteria
Population	Studies on Male Dogs Diagnosed with BPH ^2^	Papers Focusing on Other Species Besides Dogs
		Studies Focusing on Female Dogs, or Other Subgroups not Relevant to Male Dogs with BPH ^2^
Intervention	Studies Evaluating Medical Treatments for BPH ^2^, Consequently Preserving the Reproductive Function of the Male	Papers Focusing Solely on the Surgical Treatment Applied for BPH ^2^ or Treatments that Cease Sperm Production
	Studies Evaluating Treatments in both Clinical and Controlled Environments	Treatments with no Description of Dose, Duration, or Measurable Outcomes
Comparison	Studies with a Blinded Placebo or Control Group, or Single-Intervention Studies where Comparators are Available from other Studies in the Literature	Studies Without a Comparator or Relevant Literature-Based Comparator
Outcome	Studies Reporting on the Impact of BPH ^2^ Treatments on Structural or Functional Aspects of the Male Reproductive Tract	Studies that do not Report on Any Outcome Related to the Chosen Treatment
		Papers that Lack Sufficient Quantitative Data or do Not Provide Clear Outcome Measures
Other	Peer-Reviewed Manuscripts	Non-Peer-Reviewed Publications.
	Randomized Controlled Trials, Cohort Studies, Case-Control Studies, Clinical Trials, and Observational Studies	Case Reports and Small Case Series, Review Articles and Meta-Analyses, Letters to the Editor, Commentaries, and Editorials
	Studies Published in English	Other Languages
	Papers Published Between 2000–2024	Published Before the Year 2000

^1^ PICO—population, intervention, comparison, and outcome [24]. ^2^ BPH—benign prostatic hyperplasia.

## Data Availability

The original contributions presented in this study are included in the article and supplementary material. Further inquiries can be directed to the corresponding author.

## Supplementary Materials

The following supporting information can be downloaded at: https://www.mdpi.com/article/10.3390/vetsci12010070/s1, Table S1: Detailed search query used in each database; Table S2: List of papers rejected in the full text evaluation and reasons for its exclusion; Table S3: Summary of Full Text Reviewed Papers Evaluated Therapies, Followed Outcomes, Methods, and Participant Totals; Table S4: Assessment of Overall Bias Risk in the Thirty-Five Studies Selected After Full-Text Review.
